# Longevity factor klotho enhances cognition in aged nonhuman primates

**DOI:** 10.1038/s43587-023-00441-x

**Published:** 2023-07-03

**Authors:** Stacy A. Castner, Shweta Gupta, Dan Wang, Arturo J. Moreno, Cana Park, Chen Chen, Yan Poon, Aaron Groen, Kenneth Greenberg, Nathaniel David, Tom Boone, Mark G. Baxter, Graham V. Williams, Dena B. Dubal

**Affiliations:** 1 Department of Psychiatry and VA Connecticut Healthcare System, Yale School of Medicine, West Haven, CT USA; 2 Department of Neurology and Weill Institute for Neurosciences, University of California, San Francisco, CA USA; 3 Unity Biotechnology, Brisbane, CA USA; 4 Tom Boone Consulting, Newbury Park, CA USA; 5 Section on Comparative Medicine, Department of Pathology, Wake Forest University School of Medicine, Winston-Salem, NC USA

**Keywords:** Cognitive ageing, Experimental models of disease, Ageing

## Abstract

Cognitive dysfunction in aging is a major biomedical challenge. Whether treatment with klotho, a longevity factor, could enhance cognition in human-relevant models such as in nonhuman primates is unknown and represents a major knowledge gap in the path to therapeutics. We validated the rhesus form of the klotho protein in mice showing it increased synaptic plasticity and cognition. We then found that a single administration of low-dose, but not high-dose, klotho enhanced memory in aged nonhuman primates. Systemic low-dose klotho treatment may prove therapeutic in aging humans.

## Abstract (web summary)

Cognitive dysfunction in aging is a major biomedical challenge without medical therapies. Here, Castner et al. show that longevity factor klotho enhances cognition in aged nonhuman primates, increasing its relevance for a therapeutic path to humans.

## Main

Cognition is a highly valued and central manifestation of brain function eroded by aging and age-related disease such as Alzheimer’s disease. With the world’s population rapidly aging, cognitive deficits have become a major biomedical challenge in need of effective pharmacological interventions.

Klotho (KL) is a longevity factor that declines in aging^1,2^. Elevating KL boosts cognitive functions in mice through transgenic overexpression^3,4^ and acute peripheral administration^5,6^. KL (secreted α-klotho) circulates as a hormone following cleavage from its transmembrane form and impacts insulin^7^ and fibroblastic growth factor (FGF) signaling^8^, Wnt^9^ and *N*-methyl-d-aspartate receptor (NMDAR) functions^3–5^. Systemic elevation of KL in mice increases synaptic plasticity, cognition and neural resilience to aging, Alzheimer’s and Parkinson disease-related toxicities^3,10–14^. Notably, systemic administration of KL does not cross the blood–brain barrier as measured by autoradiography^15^ and His-tagged protein studies^5^. Human relevance for KL in brain health is supported by studies showing that individuals with elevated KL, due to genetic *KLOTHO* variation or other reasons, demonstrate better cognition, attenuated neuropathological measures or decreased dementia risk in aging and Alzheimer’s disease^3,16–25^.

Historically, mice have provided insights translating into important medicines^26,27^, indicating their importance in the discovery process; however, in paths to new cognitive treatments for humans, additional studies of animal models with increased genetic, anatomical and functional complexity of the brain, such as in aging nonhuman primates (NHPs), may prove critical. Whether KL treatment could enhance cognition in aging NHPs is unknown and represents a major knowledge gap.

We sought to test whether a low-dose, subcutaneous administration of KL could, in parallel to mice^5,6^, boost cognition in aged rhesus macaques, a type of NHP. Use of rhesus macaques circumvents certain limitations of mouse models, as they share 93%^28^ (versus 70%) phylogenetic similarity to humans, harbor similar or more genetic diversity than humans and demonstrate complex higher-order cognitive functions. Like humans, rhesus macaques undergo age-induced cognitive decline with synaptic changes, without significant neuronal loss, impairing brain regions, including the hippocampus^29^ and prefrontal cortex (PFC)^30^. Targeted earlier by aging^31^, the PFC subserves executive functions such as working memory and, in rhesus macaques, shows age-induced deficits in neuronal firing^32^, regulated protein kinase C (PKC) activity^33,34^, neurotransmitter balance^35,36^ and structural decline^30,37^.

Our primary goal was to test whether a KL dose in rhesus macaques that increases serum levels to a range present during the human lifespan, and is comparable to therapeutically effective increases in mice, can enhance cognition. Our secondary goal was to explore higher KL doses in rhesus macaques to test whether KL-mediated benefits on cognition could be dose-dependent.

**Fig. 1:**
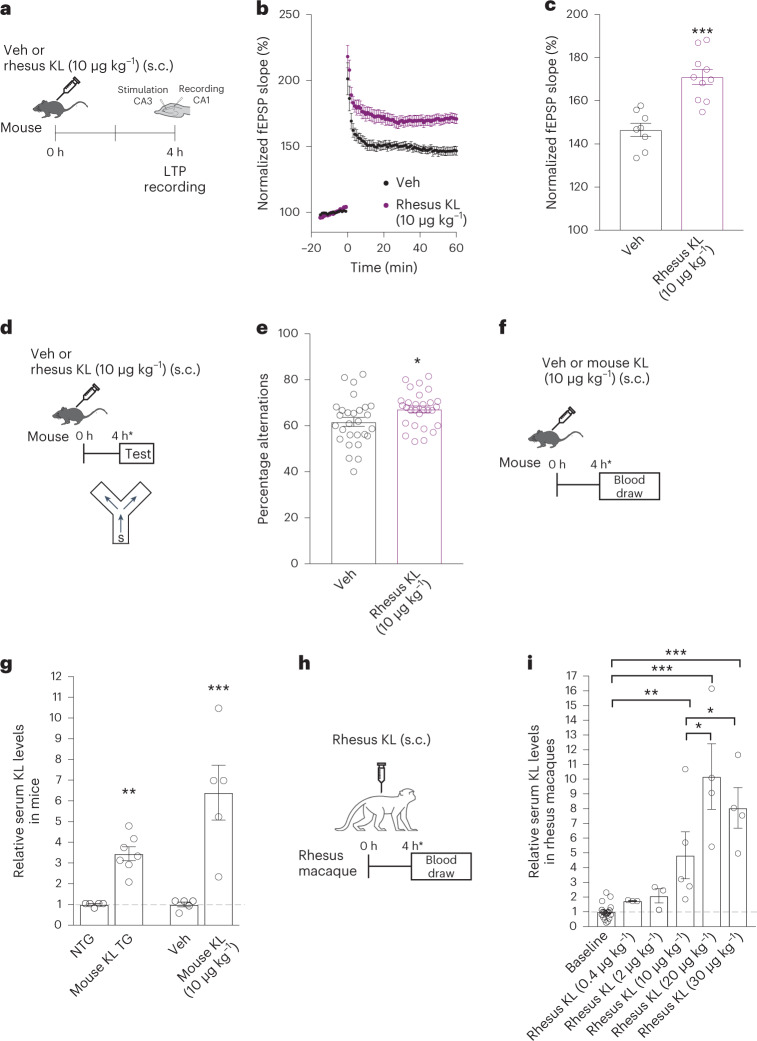
Rhesus klotho is biologically active and increases serum KL levels in aged rhesus macaques. **a**, Paradigm of hippocampal LTP recordings from young male mice treated with either vehicle (Veh) or rhesus KL (10 μg kg^−1^, s.c.). **b**, Field excitatory post-synaptic potential (fEPSP) recordings from acute hippocampal slices of mice (age 3 months) (*n* = 8 biologically independent slices from three mice for Veh; ten biologically independent slices from three mice for rhesus KL). **c**, Average fEPSP slope of the last 10 min of recordings from slices of mice (same as **b**). ***P < 0.0001 (two-tailed Student’s *t*-test); if biological unit is mouse, **P = 0.0039 (two-tailed Student’s *t*-test). **d**, Paradigm for testing male mice (age 4 months) in the small Y maze following treatment with Veh or rhesus KL (10 μg kg^−1^, s.c.). **e**, Percent alternations of mice (*n* = 28 mice, Veh; *n* = 29 mice, KL). Two independent cohorts combined. *P = 0.0243 (two-tailed Student’s *t*-test; *P = 0.0249 KL effect, accounting for cohort by linear regression two-tailed *t*-test). **f**, Paradigm for measuring serum KL levels in young male mice (age 4 months) 4 h following treatment with Veh or mouse KL (10 μg kg^−1^, s.c.). **g**, Relative KL serum levels from nontransgenic (NTG) and KL transgenic overexpressing (KL TG) mice alongside serum from young mice (age 4 months) 4 h following treatment with Veh or mouse KL (10 μg kg^−1^, s.c.) (*n* = 5 mice in each group of NTG, Veh, KL; *n* = 7 mice, KL TG). **P < 0.0001 versus NTG (two-tailed *t*-test), ***P = 0.0036 versus Veh (two-tailed *t*-test). **h**, Paradigm for measuring serum KL levels in aged rhesus macaques 4 h following treatment with Veh or varying doses of rhesus KL. **i**, Relative KL serum levels of aged rhesus macaques at baseline and following varying doses of KL (baseline, *n* = 19 monkeys; *n* = 14 female, *n* = 5 male; KL dosing, *n* = 3 monkeys in each group at 0.4 and 2 μg kg^−1^; *n* = 4 monkeys in each group at 20 and 30 μg kg^−1^; *n* = 5 monkeys at 10 μg kg^−1^; all female except one male at 10 μg kg^−1^). Values relative to baseline. *P = 0.0479 (KL 20 μg kg^−1^) and *P = 0.0508 (KL 30 μg kg^−1^) (one-tailed, one-sample *t*-tests versus KL 10 μg kg^−1^); **P = 0.0046 and ***P < 0.0001 versus baseline (two-tailed, Bonferroni-corrected). Data are represented as mean ± s.e.m. Source data

**Extended Data Fig. 1:**
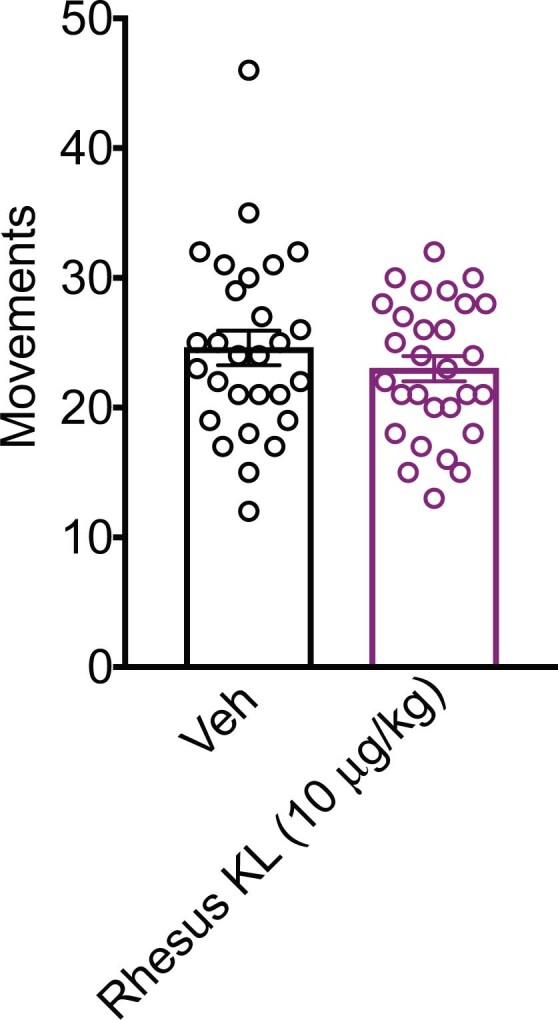
Rhesus klotho treatment of mice did not alter total arm entries in the Y maze, despite increasing % alternations. Mice (n = 28 mice, Veh; n = 29 mice, KL; age=4 months, male) treated with Veh or Rhesus KL (s.c., 10 μg/kg) did not differ in their number of entries despite klotho-mediated increased % alternations (Related to Fig. 1e). Data are mean ± SEM. Source data

We first generated the rhesus macaque form of the KL protein (96% homologous to human KL) and functionally validated its activity using assays in mice. This was the secreted, hormonal form (and not the transmembrane form) of the α-klotho protein. We tested a KL dose (10 μg kg^−1^) previously shown to increase synaptic and cognitive functions in mice^5^. As expected, 4 h after peripheral administration in mice (Fig. 1a), rhesus KL enhanced long-term potentiation (LTP), a form of synaptic plasticity (Fig. 1b,c). In parallel to LTP, rhesus KL, 4 h after peripheral administration in mice, enhanced working memory in the small Y maze (Fig. 1d,e) without altering total movements (Extended Data Fig. 1). Collectively, these data show that the rhesus form of the KL protein was biologically active in the mouse brain, with a dose previously found to be effective using mouse KL^5^.

We next assessed serum KL levels in mice with KL-mediated cognitive enhancement. We measured serum KL levels 4 h after hormone administration because peripheral KL treatment enhances cognition by that time point (Fig. 1f). Mice were treated with either vehicle or mouse KL (10 μg kg^−1^) and 4 h later their serum was collected and assayed for KL levels (Fig. 1f). Peripheral KL treatment with 10 μg kg^−1^ increased serum KL levels by approximately sixfold compared to vehicle-treated mice (Fig. 1g). As transgenic overexpression of KL also enhances cognition in mice^3,4^, we measured serum KL in transgenic mice and found 3.5-fold increases compared to nontransgenic mice (Fig. 1g). Thus, a range of 3.5- to sixfold increase of serum KL was observed in KL-mediated cognitive enhancement of mice. Whether lower levels could be efficacious is unknown.

We aimed to achieve similar increases of serum KL in aging rhesus macaques to those observed in cognitively enhanced mice. As metabolism of KL could differ between the species, we peripherally administered a range of KL doses in rhesus macaques (0.4–30 μg kg^−1^ s.c.), collected their serum 4 h later and assayed KL levels. Baseline serum KL levels were similar between aged males and females. Compared to baseline levels of KL in aging rhesus macaques, treatment with 10 μg kg^−1^ was the lowest dose tested that significantly increased serum levels by fivefold, within the desired therapeutic range observed in mice. Of note, fivefold higher levels of serum KL are observed in human cord blood compared to adults^38^ and decrease thereafter in aging^1,2^.

To execute our primary goal, we treated aged rhesus macaques (mean age ~21.78 years; Supplementary Table 1, putative human age equivalent of mean ~65 years) with a single administration of vehicle or 10 μg kg^−1^ (s.c.) of rhesus KL and tested their cognition. We chose this dose for primary analysis because it produced similar increases of KL that are (1) effective in cognitive enhancement of mice and (2) present at birth in humans^38^.

**Fig. 2:**
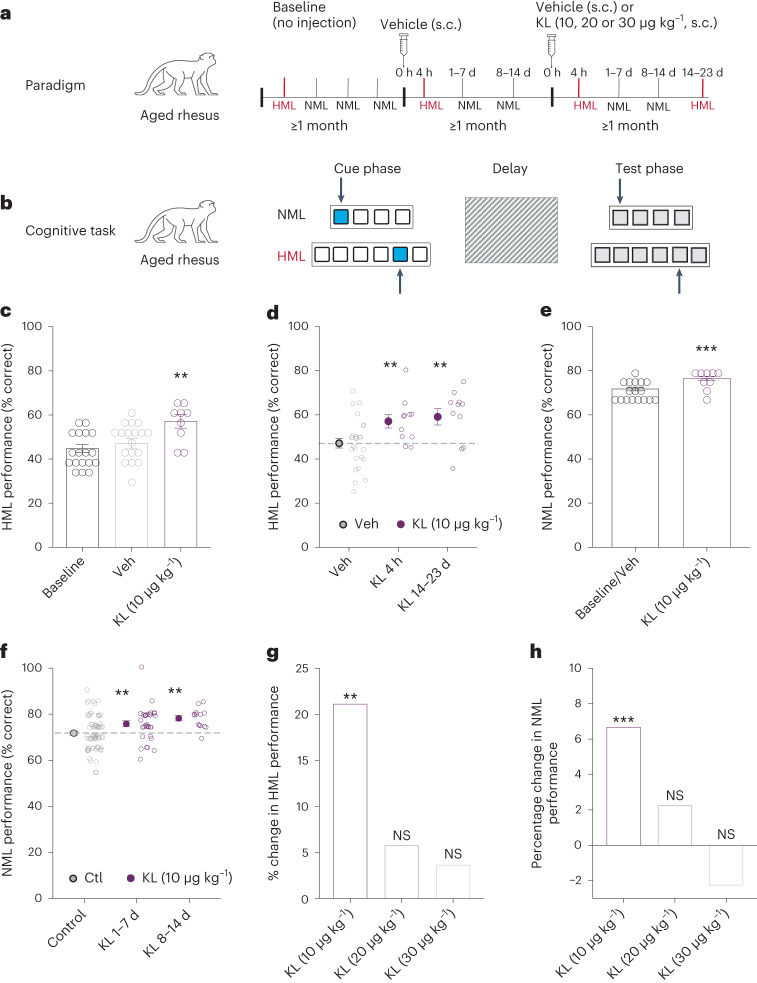
Rhesus KL enhances cognition in aged rhesus macaques. **a**, Paradigm for testing aged rhesus macaques (age 15–28 years; *n* = 18 monkeys; 13 females and 5 males) in a spatial delayed memory task following treatment with Veh or rhesus KL (Supplementary Table 1 shows testing in each monkey). **b**, Diagram of the SDR cognitive task. Monkey is shown food reward (cue phase), screen is lowered (delay) and then screen is raised with all wells covered (test phase). Remembering location of hidden reward in NML and HML (more wells and longer delays) was measured. **c**, Percent correct choices by monkeys, representing spatial and working memory, in HML task at baseline (*n* = 19 sessions from 18 monkeys; 13 females and 5 males), at 4 h following treatment with Veh (*n* = 26 sessions from 18 monkeys; 13 females and 5 males) or rhesus KL (10 μg kg^−1^) (*n* = 11 sessions from 9 monkeys; 6 females and 3 males). **P = 0.0077 versus Veh (linear mixed-model ANOVA and Satterthwaite’s *t*-tests using sessions, two-tailed). Bars represent mean ± s.e.m. of test sessions; points are mean performance of each monkey. **d**, Percent correct choices by monkeys in the HML task 4 h and 14–23 d after a single injection with Veh or rhesus KL (10 μg kg^−1^). *n*, same as Fig. 1c. **P = 0.0077 (4 h), **P = 0.0035 (14–23 d) versus Veh (linear mixed-model ANOVA and Satterthwaite’s *t*-tests using sessions, two-tailed). Points indicate sessions. Filled circles are mean ± s.e.m. of sessions. Dashed line shows mean Veh performance. **e**, Percent correct choices by monkeys in NML task 1–14 d after baseline or vehicle treatment (*n* = 71 sessions from 17 monkeys; 12 females and 5 males) or rhesus KL (10 μg kg^−1^) (*n* = 46 sessions from 9 monkeys; 6 females and 3 males), ***P = 0.0006 versus Veh + baseline (linear mixed-model ANOVA and Satterthwaite’s *t*-tests using session, two-tailed). Points show mean performance of each monkey. Bars represent mean ± s.e.m. of test sessions. **f**, Percent correct choices by monkeys in NML task averaged over the first and second weeks after baseline or Veh treatment (Ctl) (*n*, same as Fig. 1e) or rhesus KL (10 μg kg^−1^ (*n*, same as Fig. 1e; 32 sessions between 1–7 d and 14 sessions between 8–14 d), **P = 0.0080 (1–7 d) and **P = 0.0021 (8–14 d) versus Veh + baseline (linear mixed-model ANOVA and Satterthwaite’s *t*-tests using sessions, two-tailed). Points indicate sessions. Filled circles indicate mean ± s.e.m. of sessions. Dashed line shows mean control performance. **g**, Percent increase in cognition in HML task at varying doses of rhesus KL treatment at 10 μg^−1^ averaged over the course of testing (*n* = 11 sessions from 9 monkeys; 6 female and 3 male), 20 μg kg^−1^ (*n* = 7 sessions from 7 monkeys; 5 female and 2 male) or 30 μg kg^−1^ (*n* = 13 sessions from 13 monkeys; 9 females and 4 males) compared to Veh treatment (*n* = 26 sessions from 18 monkeys; 13 females and 5 males). **P = 0.0077 versus Veh (linear mixed-model ANOVA and Satterthwaite’s *t*-tests using sessions, two-tailed). **h**, Percent increase in cognition in NML task at varying doses of rhesus KL treatment at 10 μg kg^−1^ averaged over the course of testing (*n* = 46 sessions from 9 monkeys; 6 female and 3 male), 20 μg kg^−1^ (*n* = 31 sessions from 7 monkeys; 5 female and 2 male) or 30 μg kg^−1^ (*n* = 40 sessions from 13 monkeys; 9 females and 4 males) compared to Veh + baseline (*n* = 71 sessions from 17 monkeys; 12 females and 5 males) ***P = 0.0006 versus Veh (linear mixed-model ANOVA and Satterthwaite’s *t*-tests using sessions, two-tailed). Source data

Cognitive testing of aged rhesus macaques was performed using the spatial delayed response (SDR) task (Fig. 2a), assessing frontal-temporal circuits and regions of the brain, including the hippocampus and PFC. This task assesses working and spatial memory for both a normal memory load (NML; easier task) and high memory load (HML; harder task)^39^ (Fig. 2b). SDR is an ideal task to test KL because aging disrupts the specific cognitive domains that it probes^31,40^. In brief, monkeys were trained to achieve a stable baseline response for the NML task in remembering the spatial location of a food reward (Fig. 2a). All macaques were then treated with vehicle (s.c.) to habituate to effects of the procedure and stress of an injection on cognitive performance. Finally, monkeys were treated with either vehicle or rhesus KL (s.c.); 4 h later, monkeys underwent the HML task (with up to nine wells) followed by a series of NML tasks (up to seven wells) over 2 weeks (Fig. 2a), ending with another HML task.

In the primary analysis, KL (10 μg kg^−1^, s.c.) enhanced cognition in aged rhesus macaques during both NML and HML testing. As expected, performance between baseline or vehicle treatment did not differ. KL (10 μg kg^−1^, s.c.) increased HML performance by 4 h after treatment (Fig. 2c), within the same rapid time frame it increased cognition in mice (Fig. 1e). KL-mediated cognitive enhancement of HML, a harder memory task, persisted at 2 weeks (Fig. 2d). KL (10 μg kg^−1^, s.c.) also enhanced the average NML performance (Fig. 2e), an effect that persisted across multiple tests during the first and second weeks following treatment (Fig. 2f). KL-mediated enhancement was observed independent of sex.

In exploratory analysis of higher KL doses (20 and 30 μg kg^−1^, s.c.), significant cognitive improvement was not observed in either the HML (Fig. 2g) or the NML (Fig. 2h) tasks. It is interesting to note that in contrast to monkeys, mice continued to show cognitive enhancement with much higher KL doses^5^. This species difference may be related to increased structural and network complexity of the monkey compared to the mouse brain.

**Extended Data Fig. 2:**
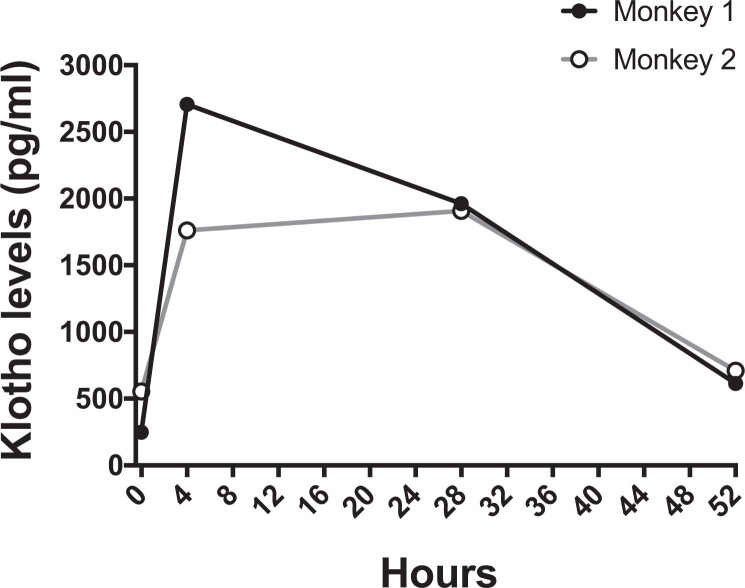
Time course of serum klotho levels following treatment of two aged rhesus macaques. Following Rhesus KL injection (s.c., 30 μg/kg), blood was collected at 4 h, 28 h and 52hrs (n = 8 samples from 2 monkeys, female, ages 22 and 24 years). Assuming peak concentration at 4 h, putative klotho half-life was 29.5 h with predicted elimination in 6.15 days. Source data

Collectively, our data show that KL (10 μg kg^−1^) enhanced cognition in aging rhesus macaques, an effect that persisted for at least 2 weeks in both the NML and HML measures of memory. KL-mediated cognitive enhancement similarly persisted in mice for at least 2 weeks^5^, suggesting organizational, longer-lasting and beneficial effects on the synapse and brain. In both species, the cognitive effect outlasted the putative half-life of the hormone, 7 min^15^ in rodents and estimated at 29.5 h in aging rhesus macaques (*n* = 8 samples from two monkeys; Extended Data Fig. 2). KL induced a greater change in HML compared to NML, potentially because HML is a harder and more sensitive task with more dynamic range for improvement.

Higher doses of KL (20 and 30 μg kg^−1^) did not enhance cognition in monkeys. Of note, the higher doses tested did not impair cognition as the 2–5% changes were not significantly increased or decreased statistically; however, it remains to be determined whether doses even higher than those tested could impair cognition. Together, these data indicate that KL-mediated cognitive enhancement extends to NHPs in a complex genetic, anatomical and functional brain similar to humans. These data also suggest that lower, more ‘physiological’ levels of the hormone in the body may be required for a therapeutic window of cognitive enhancement in humans.

As KL has pleiotropic actions, including on insulin^7^ and FGF signaling^8^, Wnt^9^ and NMDAR functions^3–5^, it is interesting to speculate that the specificity of its action at low doses represents a balanced, multimodal effect across signaling systems that inherently benefits biological substrates of cognition. Higher doses of KL, beyond what is experienced over the human lifespan, could differentially impact signaling systems to create imbalances that no longer enhance cognition. Whether even lower doses of KL than those tested could also enhance cognition remains to be determined. Further, because peripherally injected KL does not cross into the brain^5,15^, peripheral messengers that transduce its signals into the brain should be identified.

Potential limitations of our study include that monkeys were tested on one cognitive task (SDR) and were not drug naive, though their cognitive testing was confirmed to have stably returned to baseline. We chose SDR because it is sensitive to aging and measures functions of the hippocampus and the PFC, a cognitive hub enhanced in individuals with a *KLOTHO* variant leading to higher systemic KL levels^25^. SDR, which does not exhibit practice effects because each trial is unique (combined with a longitudinal experimental design), enabled testing of multiple time points after KL treatment using comparable testing procedures.

Because KL levels decrease in human aging^1,2^, our data showing that a lower dose of KL (comparable to five times baseline levels and similar to levels observed at birth^38^) can enhance cognition in aged NHPs suggest that peripheral treatment or replenishment with this endogenous hormone may prove therapeutic in aging humans.

## Methods

### Animals

All mice were on a congenic C57BL/6J background and kept on a 12-h light–dark cycle with ad libitum access to food and water. The ambient temperature was approximately 70 °F and humidity was 42–43%. The standard housing group was five mice per cage. Behavioral tasks were carried out during the light cycle. All studies were approved by the Institutional Animal Care and Use Committee of the University of California, San Francisco, and conducted in compliance with National Institutes of Health guidelines. Adult aged male and female rhesus macaque monkeys (*Macaca* *mulatta*) were on average (s.d.) 21.78 (3.80) years for monkeys ranging from 15–28 years; Supplementary Table 1). The putative human equivalent using a 1:3 conversion is 65.33 (11.39) years, ranging 45–84 years. Monkeys were maintained in accordance with the Yale University Institutional Animal Care and Use Committee and federal guidelines for the care and use of nonhuman primates. Monkeys were maintained in a 12-h light–dark cycle, fed standard monkey chow and fruit and were tested during the light cycle.

### Drug treatment

Mouse KL and rhesus KL were diluted in PBS (pH 7.5) and injected s.c. at specified doses before behavior testing or sample collection for serum KL measurements. Recombinant proteins were used within 1 week of thawing from −80 °C stock solutions and stored at 4 °C. Proteins were coded to keep experimenters blind to the identity of treatment used in experiments for all mouse studies and for the majority of monkey studies performed.

### Serum KL measurements

ELISAs was performed to measure KL from monkey serum samples, according to the manufacturer’s directions as described^3^. Briefly, each monkey serum sample was diluted with ELISA buffer (1:2) and analyzed for KL by ELISA. One serum value, several s.d. above the cohort mean was excluded from analysis. For mouse serum, samples were assayed using immunoprecipitation-immunoblot for mouse KL by the O’Brien Kidney Research Core at UT Southwestern as described elsewhere^41^.

### Electrophysiology

Mouse brain slices (300 μm) were obtained from coronal sections as described with some modifications^3–5^ from the hippocampal CA1 region following Schaffer collateral path stimulation. Briefly, mice were anesthetized, the brain was placed in ice-cold artificial cerebrospinal fluid, sliced on a vibratome (Leica VT1200) and slices were incubated (32 °C for 30 min, then room temperature for 1 h before testing). Hippocampal slices were positioned on a Med64-Quad II multielectrode array (Alpha MED Scientific). Using planar electrodes (Quad II 2 × 8 Probe AL-MED-PG501A), the fEPSPs were produced and recorded. Using a theta-burst protocol, LTP was generated with a theta-burst protocol. Analysis and recordings were executed with Med64 Mobius Software (Alpha MED Scientific). After a stable induction of LTP, the final 10 min of a 60-min recording were averaged for each experimental group.

### Small Y maze

Mice were tested in the small Y maze, also known as spontaneous alternation Y maze with spatial cues, as described^3–5^. Briefly, a mouse was placed inside one of the three identical arms of the Y maze and allowed to explore the apparatus for 6 min. The recorded spontaneous alterations (Ethnovision XT v.10, Noldus) were scored manually and percent alternations were calculated. Two mouse cohorts were tested at the same time of day.

### Spatial delayed response task

The SDR task with NML and HML was performed as described previously^39,42^. Briefly, following training to stability (65–75% correct) on the NML task, monkeys were tested at approximately the same time of day for highly palatable food rewards. Stable performance was first achieved for each monkey by titrating task difficulty by increasing the number of spatial locations or lengthening the delays (a variable multiplier between one and ten applied to a set of baseline delays of 0, 1, 2, 3 and 4 s). Once stable, the number of wells and delay multiplier was kept fixed for that monkey for the duration of the testing for the current study. For testing, monkeys watched the spatially distanced wells while the investigator put a preferred treat in a single well. The wells were covered, an opaque screen between the monkey and wells was lowered and after a predetermined delay, the screen was raised. The monkey was able to retrieve the treat if the choice of well was correct. Based on the number of wells and the time between cue and task the behavior is classified as either HML (6–9 wells; 0–15-s delay) or NML (4–7 wells; 0–32-s delay).

All animals had extensive training and testing on the task, typically over many years and participated in previous studies. During that time they exhibited stability in their working memory performance (70 ± 2.5% s.e.m. correct) on multiple occasions, including immediately before commencing engagement in the current study. Animals performed 20 trials on any one test session, with generally one test session performed on any given day. A typical testing session lasted less than 10 min. In nearly all cases, animals were tested on a minimum of four SDR sessions between HML sessions to ensure stability or to detect any long-term changes in performance. A total of 13 animals were tested at the dose of 30 µg kg^−1^, 7 at 20 µg kg^−1^ and 9 at 10 µg kg^−1^ (two of them twice). One animal only received vehicle. Monkeys were used again to test other treatments such as vehicle or other KL doses only after cognitive testing was confirmed to be at the monkey’s baseline. The history of testing for each monkey is provided in Supplementary Table 1.

### Statistics and reproducibility

Statistical analyses were carried out with GraphPad Prism (v.7.0) for *t*-tests and analysis of variance (ANOVA). Two-tailed *t*-tests were used to determine differences between two means and two-way ANOVA was used to determine differences between multiple means. Post hoc corrections were performed for multiple comparisons as indicated. Linear mixed-model analysis for determining the effect of KL on task performance was performed with lme4 in R^43^ with drug condition as a fixed effect and monkey as a random effect, accounting for repeated testing of some monkeys across test conditions. For analyses, baseline and vehicle HML sessions and post-baseline/vehicle NML test sessions were only used when monkeys were naive to KL treatment. Error bars represent ±s.e.m. Null hypotheses were rejected at or below a *P* value of 0.05. For data exclusion, for mouse studies, exclusion criteria (greater than 2 s.d. above or below the mean) were defined a priori to ensure unbiased exclusion of outliers. In monkey studies, a monkey was excluded if it refused to perform the behavior task. No randomization method was used to allocate animals to experimental groups. Sample sizes were determined by assessing data reported in previous publications on KL in mice^5,6^ and in pharmacological studies of rhesus macaques^44^. Data distribution was normal in studies of mouse synaptic plasticity and behaviors studies. For linear mixed-model analyses of monkey studies, residual plots were inspected for evidence of violation of assumptions; none was apparent. Blinding was used for mouse studies of behavior and synaptic plasticity and for the majority (>50%) of monkey behavior test sessions that included baseline, vehicle and varying KL doses (unblinded data did not differ appreciably from blinded data). Blinding was not used for ELISA studies.

### Reporting summary

Further information on research design is available in the Nature Portfolio Reporting Summary linked to this article.

## Supplementary information

Supplementary Information Supplementary table 1.

Reporting Summary

## Data Availability

All data supporting the findings of this study are available within the source data provided with this paper.

## Source data

Source Data Fig. 1 Statistical source data.
Source Data Fig. 2 Statistical source data.
Source Data Extended Data Fig.1 Statistical source data.
Source Data Extended Data Fig. 2 Statistical source data.

## Code availability

There was no new code created for analysis of these data.
