# Supplementary material for: Longevity factor klotho enhances cognition in aged nonhuman primates
Source: Nat Aging. 2023 Jul 3;3(8):931–7. doi: 10.1038/s43587-023-00441-x (PMC10432271; doi:10.1038/s43587-023-00441-x)
Supplement: Supplementary file 1 — Supplementary table 1. [file 43587_2023_441_MOESM1_ESM.pdf]

nature aging

Brief Communication

https://doi.org/10.1038/s43587-023-00441-x

Longevity factor klotho enhances cognition in aged nonhuman primates

In the format provided by the authors and unedited

Supplementary Table 1. Monkey details and sequence of testing in each case (monkey). Monkeys were only used again to test other treatments like Vehicle or other klotho doses after cognitive testing was confirmed to be at the monkey's baseline.

**Case**	**Sex**	**Age**	**Sequence of test sessions**
1	F	26yrs	baseline, vehicle, KL 30, vehicle, KL 20, baseline, vehicle, baseline
2	F	23yrs	baseline, vehicle, vehicle, KL 30, KL 10, baseline, KL 10, baseline
3	F	18yrs	baseline, vehicle, vehicle, KL 30, KL 10, baseline, baseline
4	M	17yrs	baseline, vehicle, KL 30, vehicle, KL 20, baseline, baseline
5	F	24yrs	baseline, vehicle, KL 30, vehicle, KL 20, baseline, baseline
6	F	26yrs	baseline, vehicle, KL 30, vehicle, KL 10, baseline, baseline, vehicle, baseline
7	M	19yrs	baseline, vehicle, vehicle, KL 10, baseline
8	M	22yrs	baseline, vehicle, vehicle, KL 30, KL 10, baseline, baseline, KL 10, baseline
9	M	23yrs	baseline, vehicle, KL 30, vehicle, KL 10, baseline, baseline, vehicle, baseline
10	F	23yrs	baseline, vehicle, KL 10, baseline, baseline
11	F	27yrs	baseline, vehicle, vehicle, KL 30, baseline, KL 20, baseline
12	F	19yrs	baseline, vehicle, vehicle, KL 30, baseline
13	F	19yrs	baseline, vehicle, KL 30, vehicle, baseline, KL 20, baseline
14	F	18yrs	baseline, vehicle, KL 30, baseline, KL 20
15	M	25yrs	baseline, vehicle, vehicle, KL 30, baseline, KL 20, baseline
16	F	20yrs	baseline, vehicle, KL 10, baseline
17	F	15yrs	baseline, vehicle, KL 10, baseline
18	F	28yrs	baseline, vehicle, vehicle, baseline
