# Supplementary material for: Longevity factor klotho enhances cognition in aged nonhuman primates
Source: Nat Aging. 2023 Jul 3;3(8):931–7. doi: 10.1038/s43587-023-00441-x (PMC10432271; doi:10.1038/s43587-023-00441-x)
Supplement: Supplementary file 2 — Reporting Summary [file 43587_2023_441_MOESM2_ESM.pdf]

Corresponding author(s): Dena Dubal

Last updated by author(s): May 9, 2023

Reporting Summary

Nature Portfolio wishes to improve the reproducibility of the work that we publish. This form provides structure for consistency and transparency in reporting. For further information on Nature Portfolio policies, see our Editorial Policies and the Editorial Policy Checklist.

Statistics

For all statistical analyses, confirm that the following items are present in the figure legend, table legend, main text, or Methods section.

n/a	Confirmed	
☐	☒	The exact sample size (*n*) for each experimental group/condition, given as a discrete number and unit of measurement
☐	☒	A statement on whether measurements were taken from distinct samples or whether the same sample was measured repeatedly
☐	☒	The statistical test(s) used AND whether they are one- or two-sided<br>*Only common tests should be described solely by name; describe more complex techniques in the Methods section.*
☒	☐	A description of all covariates tested
☐	☒	A description of any assumptions or corrections, such as tests of normality and adjustment for multiple comparisons
☐	☒	A full description of the statistical parameters including central tendency (e.g. means) or other basic estimates (e.g. regression coefficient) AND variation (e.g. standard deviation) or associated estimates of uncertainty (e.g. confidence intervals)
☐	☒	For null hypothesis testing, the test statistic (e.g. *F*, *t*, *r*) with confidence intervals, effect sizes, degrees of freedom and *P* value noted<br>*Give P values as exact values whenever suitable.*
☒	☐	For Bayesian analysis, information on the choice of priors and Markov chain Monte Carlo settings
☒	☐	For hierarchical and complex designs, identification of the appropriate level for tests and full reporting of outcomes
☒	☐	Estimates of effect sizes (e.g. Cohen's *d*, Pearson's *r*), indicating how they were calculated

Our web collection on statistics for biologists contains articles on many of the points above.

Software and code

Policy information about availability of computer code

Data collection	Med64 Mobius Software (Alpha MED Scientific Inc), Ethovision XT (version 10, Noldus)
Data analysis	For monkey behavior data, linear mixed model analysis for determining effect of klotho on task performance was performed with R (lme4). For mouse LTP, small Y maze, mouse and monkey ELISAs, statistical analyses were carried out with GraphPad Prism (version 7.0) for t-tests and analyses of variance (ANOVAs). Two-tailed t-tests were used to determine differences between two means and two-way ANOVA was used to determine differences between multiple means unless indicated otherwise. Post-hoc corrections were performed for multiple comparisons as indicated.

For manuscripts utilizing custom algorithms or software that are central to the research but not yet described in published literature, software must be made available to editors and reviewers. We strongly encourage code deposition in a community repository (e.g. GitHub). See the Nature Portfolio guidelines for submitting code & software for further information.

Data

Policy information about availability of data

All manuscripts must include a data availability statement. This statement should provide the following information, where applicable:

- Accession codes, unique identifiers, or web links for publicly available datasets
- A description of any restrictions on data availability
- For clinical datasets or third party data, please ensure that the statement adheres to our policy

Data supporting the findings of this study are available within the source data provided with this paper.

Field-specific reporting

Please select the one below that is the best fit for your research. If you are not sure, read the appropriate sections before making your selection.

☒ Life sciences ☐ Behavioural & social sciences ☐ Ecological, evolutionary & environmental sciences

For a reference copy of the document with all sections, see nature.com/documents/nr-reporting-summary-flat.pdf

Life sciences study design

All studies must disclose on these points even when the disclosure is negative.

Sample size	Typical sample sizes in our studies of synaptic plasticity and behavior with klotho are chosen based on previous experiments and studies (PMID: 35428698, 28793260). This includes: synaptic plasticitv in mice (n=3-6 mice per group), behavior in mice (n=15-30 per group), and behavior in monkeys (n= 10-20 per group). For ELISA klotho level studies, sample sizes of n=3-7 were considered required to yield biologically relevant differences; this was considered sufficient since pilot studies showed several fold increases for klotho levels with transgenic overexpression or treatment in mice. For monkey ELISA klotho level studies, we were limited to monkeys that were available for injection and blood draw.
Data exclusions	In mouse studies, exclusion criteria (greater than 2 SDs above or below the mean) were defined a priori to ensure unbiased exclusion of outliers. In monkey studies, the monkey was excluded if it refused to perform the behavior task.
Replication	Significant results in the small Y maze of mice were tested again and successfully replicated using rhesus klotho. In other experiments, klotho-enhancing LTP results were replicated over five times using mouse and human forms of klotho (data not shown). All attempts at replication were successful. ELISA studies and monkey behavior studies were not replicated in independent cohorts.
Randomization	Allocation to groups was random.
Blinding	In mouse studies, experimenters were blinded to the treatment of all mice. In monkey studies, experimenters were blinded to treatment in the majority (>50%) of monkey behavior test sessions that included baseline, vehicle, and varying klotho doses . Blinded data did not appreciably differ from unblinded test behavior data. Monkey ELISA studies were not blinded since only klotho was injected for determining klotho level increase.

Reporting for specific materials, systems and methods

We require information from authors about some types of materials, experimental systems and methods used in many studies. Here, indicate whether each material, system or method listed is relevant to your study. If you are not sure if a list item applies to your research, read the appropriate section before selecting a response.

Materials & experimental systems

n/a	Involved in the study	
☒	☐	Antibodies
☒	☐	Eukaryotic cell lines
☒	☐	Palaeontology and archaeology
☐	☒	Animals and other organisms
☒	☐	Human research participants
☒	☐	Clinical data
☒	☐	Dual use research of concern

Methods

n/a	Involved in the study	
☒	☐	ChIP-seq
☒	☐	Flow cytometry
☒	☐	MRI-based neuroimaging

Animals and other organisms

Policy information about studies involving animals; ARRIVE guidelines recommended for reporting animal research

Laboratory animals	All mice were on a congenic C57BL/6J background and were male (3-4 months old). Male and female monkeys were tested as described in the figure legends. Monkeys were rhesus macaques (Macaca mullata) (15-28 years old).
Wild animals	The study did not involve wild animals
Field-collected samples	The study did not involve samples collected from the field
Ethics oversight	All mouse studies were approved by the Institutional Animal Care and Use Committee of the University of California, San Francisco, and conducted in compliance with NIH guidelines. All monkey studies were approved by Yale University Institutional Animal Care and Use Committee

Note that full information on the approval of the study protocol must also be provided in the manuscript.
